# Novel EGFR inhibitors attenuate cardiac hypertrophy induced by angiotensin II

**DOI:** 10.1111/jcmm.12763

**Published:** 2016-01-14

**Authors:** Kesong Peng, Xinqiao Tian, Yuanyuan Qian, Melissa Skibba, Chunpeng Zou, Zhiguo Liu, Jingying Wang, Zheng Xu, Xiaokun Li, Guang Liang

**Affiliations:** ^1^ Chemical Biology Research CenterSchool of Pharmaceutical ScienceWenzhou Medical UniversityWenzhouZhejiangChina; ^2^ Department of UltrasonographyThe 2^nd^ Affiliated Hospital of Wenzhou Medical UniversityWenzhouZhejiangChina

**Keywords:** angiotensin II, epidermal growth factor receptor, cardiac hypertrophy, c‐Src, small‐molecule inhibitor

## Abstract

Cardiac hypertrophy is an important risk factor for heart failure. Epidermal growth factor receptor (EGFR) has been found to play a role in the pathogenesis of various cardiovascular diseases. The aim of this current study was to examine the role of EGFR in angiotensin II (Ang II)‐induced cardiac hypertrophy and identify the underlying molecular mechanisms. In this study, we observed that both Ang II and EGF could increase the phospohorylation of EGFR and protein kinase B (AKT)/extracellular signal‐regulated kinase (ERK), and then induce cell hypertrophy in H9c2 cells. Both pharmacological inhibitors and genetic silencing significantly reduced Ang II‐induced EGFR signalling pathway activation, hypertrophic marker overexpression, and cell hypertrophy. In addition, our results showed that Ang II‐induced EGFR activation is mediated by c‐Src phosphorylation. *In vivo*, Ang II treatment significantly led to cardiac remodelling including cardiac hypertrophy, disorganization and fibrosis, accompanied by the activation of EGFR signalling pathway in the heart tissues, while all these molecular and pathological alterations were attenuated by the oral administration with EGFR inhibitors. In conclusion, the c‐Src‐dependent EGFR activation may play an important role in Ang II‐induced cardiac hypertrophy, and inhibition of EGFR by specific molecules may be an effective strategy for the treatment of Ang II‐associated cardiac diseases.

## Introduction

Cardiovascular diseases (CVDs) remain the leading causes of death and disability in the world. Although a large proportion of CVDs is preventable, their incidence continues to rise mainly because preventive measures are inadequate 1. During the pathological development of CVDs, cardiac hypertrophy plays a critical role. Cardiac hypertrophy is an adaptive response of the heart that occurs in various CVDs, when the heart responds to a variety of extrinsic and intrinsic stimuli that impose increased biomechanical stress. Although hypertrophy can eventually normalize wall tension, prolonged hypertrophy typically culminates in chronic heart failure or sudden cardiac death 2. Understanding the mechanisms and potential targets underlying cardiac hypertrophy is thus important to the field of cardiovascular biology, and may lead to new strategies for the prevention or treatment of CVDs.

The renin–angiotensin system plays a vital role in regulating the physiological and pathological responses of cardiovascular system. Its primary effector hormone, angiotensin II (Ang II), not only mediates immediate physiological effects of vasoconstriction and blood pressure regulation but is also implicated in inflammation, endothelial dysfunction, atherosclerosis, hypertension and cardiac hypertrophy in a series of CVDs 3, 4, 5. Many studies support the observation that Ang II has direct effects on myocardial cells, including cardiomyocyte growth, accumulation of extracellular matrix and hypertrophy 4, 6, 7. These effects are known to be mediated by angiotensin type 1 receptor (AT1R), demonstrated by the fact that AT1R antagonists prevent Ang II‐induced cardiac hypertrophy in rats 8. A variety of downstream signalling cascades have been found to be involved in mediating Ang II's pathological effects. In addition to the classic G protein‐mediated pathways, Ang II has been reported to activate several protein kinases, including serine/threonine and receptor tyrosine kinases 9. Recent studies using targeted overexpression of AT1R in cardiomyocytes suggest that Ang II can directly promote the growth of cardiomyocytes *via* transactivation of epidermal growth factor receptor (EGFR) and subsequent activation of mitogen‐activated protein kinases (MAPKs) 10.

Epidermal growth factor receptor, also known as ErbB1, is a receptor tyrosine kinase and belongs to the ErbB family. When its ligands, EGF and heparin bound‐EGF, bind to a single receptor, conformational changes occur which allow dimerization and allosteric activation of the tyrosine kinase in the cytoplasm 11. The phosphorylation of EGFR consequently recruits adapter signalling molecules such as AKT and ERK. Classically, EGFR is widely acknowledged for its influence in tumour biology and wound healing and at least six EGFR‐specific inhibitors have been used in clinical cancer therapy 12. However, an additional role of EGFR in maintaining organ and cellular homoeostasis is becoming more and more evident in recent years, especially in the endocrinology and cardiovascular system 13, 14. Recently, EGFR inhibition by small‐molecule inhibitors has been demonstrated to be able to attenuate insulin resistance, atherosclerosis and diabetic microvascular complications 13, 15, 16.

**Figure 1:**
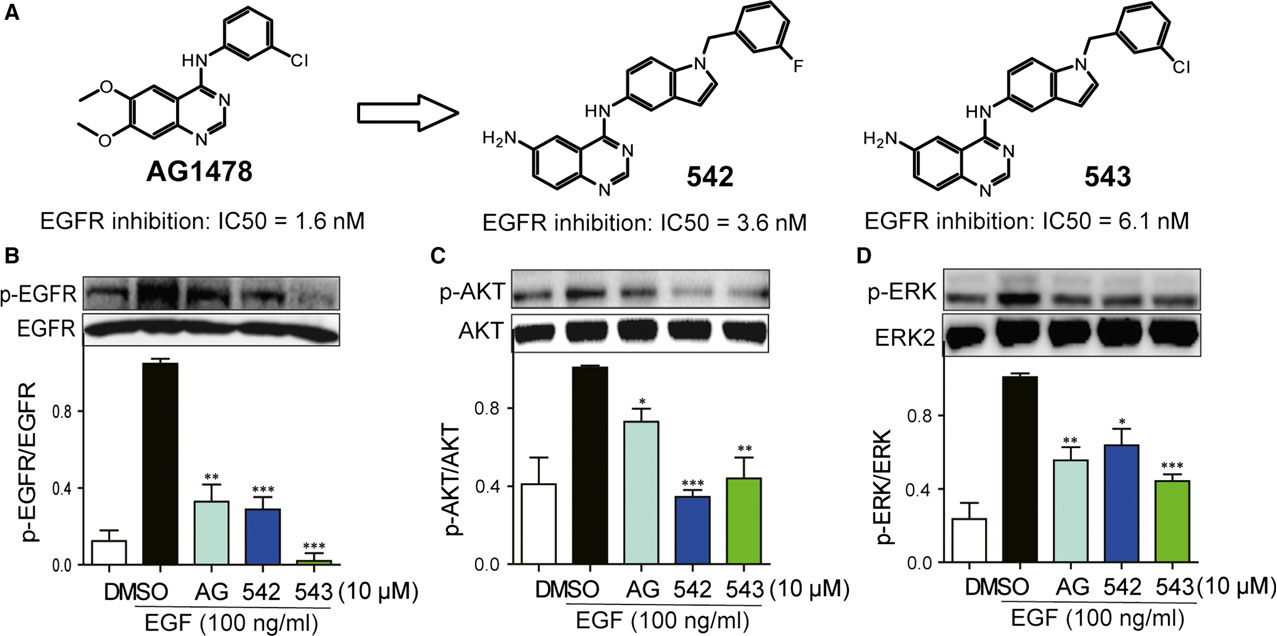
Small‐molecule inhibitors inhibited EGFR activation in H9c2 cells. (**A**) Chemical structures of AG1478, 542 and 543 with their IC_50_s against EGFR kinase activity. (**B**–**D**) H9c2 cells were pre‐treated with AG1478 (10 μΜ), 542 (10 μΜ) or 543 (10 μΜ) for 2 hrs, and followed incubation of EGF (100 ng/ml) for 15 min. The cell lysates were collected and p‐EGFR/EGFR (**B**), p‐AKT/AKT (**C**) and p‐ERK/ERK (**D**) were detected by Western blot analysis. The columns show the normalized optical density for data from three independent experiments. *P < 0.05, **P < 0.01, ***P < 0.001 *versus* EGF group.

AG1478 is a well‐published EGFR‐specific inhibitor and is widely used in EGFR‐related biological studies 17. Our group has been engaged in the medicinal chemistry and drug discovery of receptor tyrosine kinase inhibitors for years. We previously designed and synthesized a series of AG1478 analogues as EGFR inhibitors. Among these analogues, compounds 542 and 543 exhibited strong and selective EGFR‐inhibitory activity at both the molecular and cellular levels, with the IC_50_ of 3.6 and 6.1 nM against recombinant EGFR kinase activity respectively (Fig. 1A). The aim of this study was to test if the novel EGFR inhibitors are able to attenuate Ang II‐induced cardiac hypertrophy both *in vitro* and *in vivo* and identify the underlying mechanism.

## Materials and methods

### Materials

AngII and AG1478 were purchased from Sigma‐Aldrich (St. Louis, MO, USA). FITC‐Phalloidin was purchased from Enzo (Enzo, New York, NY, USA). Epidermal growth factor was purchased from R&D (R&D Biotech Ltd., Minneapolis, MN, USA). Epidermal growth factor receptor shRNA plasmid was purchased from Santa Cruz Biotech (Santa Cruz, CA, USA). Compounds 542 and 543 were synthesized and characterized by our laboratory(purity: 98.6% and 99.1% respectively) and their EGFR kinase inhibitory activities were detected by Caliper Mobility Shift Assay (Fig. 1A). The compounds were dissolved in Dimethyl Sulphoxide (DMSO) for *in vitro* experiments and were dissolved in 1% CMC‐Na for *in vivo* experiments. The antibodies for p‐AKT^Ser473^ (sc33437), AKT (sc1619), p‐EGFR^Tyr1173^ (sc12351), EGFR (sc31155), p‐ERK1/2 (sc7383), ERK (sc292838), p‐c‐Src (sc16846), c‐Src (sc8056), MyHC (sc20641) and GAPDH (sc25778) were purchased from Santa Cruz Biotech.

### Cell culture

The immortalized rat cardiomyocyte cell line H9c2 was obtained from the American Type Culture Collection (ATCC, Manassas, VA, USA). The cells were cultured in DMEM/F12 supplemented with 10% foetal bovine serum, 100 U/ml penicillin and 100 U/ml streptomycin at 37°C in a humidified 5% CO_2_ atmosphere.

### Transient transfection of EGFR shRNA

The small hairpin RNA (shRNA) specifically targeting the nucleotides of EGFR and its control shRNA contained in the plasmid were obtained from Santa Cruz Biotech. H9c2 cells were transiently transfected with shRNAs (1 μg/ml) using PolyJet transfection reagent (SignaGen Laboratories, Ljamsville, MD, USA) at a ratio of 3 μl PolyJet to 1 μg plasmid in DMEM/F12 containing 10% fetal bovine serum (FBS) for 48 hrs according to the manufacturer's instructions. Briefly, H9c2 cells were trypsinized, counted, plated at a density of 5 × 10^4^ cell in 35 mm dishes in antibiotic‐free DMEM/F12 with 10% FBS, and incubated overnight at 37°C with 5% CO_2_. The next morning, the medium was changed, and ~60–70% confluent cells were transfected with 1 μg plasmid of EGFR shRNA or control shRNA using 3 μl PolyJet/well. After 48 hrs, cell lysates were subjected to Western blot and RT‐qPCR analysis.

### Immunofluorescence staining

Cardiomyocytes cultured on coverslips were washed with PBS, fixed in a 4% paraformaldehyde solution in PBS for 10 min. at room temperature, permeabilized with 0.1% (v/v) Triton X‐100 in PBS for 5 min. at room temperature and then blocked with 5% bovine serum albumin (BSA) for 0.5 hr at room temperature. Cells were incubated with FITC‐Phalloidin (5 μg/ml) for 1 hr. After incubating cells with the 4',6‐diamidino‐2‐phenylindole (DAPI) nuclear stain, coverslips were mounted with antifading mounting media (Invitrogen, Carlsbad, CA, USA), and images were captured at the same magnification (×60) on a FV10i confocal microscope and processed by FV10i software (Olympus, Tokyo, Japan).

### Animal experiments

All animal care and experimental procedures complied with the ‘The Detailed Rules and Regulations of Medical Animal Experiments Administration and Implementation’ (Order no. 1998‐55, Ministry of Public Health, China.), and ‘Ordinance in Experimental Animal Management’ (Order no. 1998‐02, Ministry of Science and Technology, China) and were approved by the Wenzhou Medical College Animal Policy and Welfare Committee (Approval Document no. 2012/ACWC/0204). Male C57BL/6 mice (*n* = 35) weighing 18–22 g aged 4 weeks were obtained from the Animal Centre of Wenzhou Medical University (Wenzhou, China). Animals were housed with a 12:12 hrs light–dark cycle at a constant room temperature, fed a standard rodent diet and free accessed to water. The animals were acclimatized to the laboratory for at least 2 weeks before experiment.

C57BL/6 mice were randomly divided into five groups with seven mice in each group: (*i*) non‐treated vehicle control mice (control group); (*ii*) AngII‐induced cardiac hypertrophy mice that received PBS alone (Ang II group); (*iii*) AngII‐induced cardiac hypertrophy mice that were treated subcutaneous with AG1478 (in 1% CMC‐Na solution) at a dose of 20 mg/kg/day starting at day 1 before AngII injection for 2 weeks (AngII + AG1478 group); (*iv*) AngII + 542 5.0 mg/kg/day group; and (*v*) AngII+ 542 20.0 mg/kg/day group. Cardiac hypertrophy was induced in 6‐week‐old C57BL/6 mice by a single subcutaneous injection of AngII (0.5 mg/kg/2 days for 2 weeks) in phosphate buffer (pH 7.2), as described previously 18. Mice in vehicle control group and Ang II group received 1% CMC‐Na solution alone in the same schedule as compound‐treated groups. Animals were killed under sodium pentobarbital anaesthesia. After mice was killed, heart tissues were embedded in 4% paraformaldehyde for pathological analysis and/or snap‐frozen in liquid nitrogen for gene and protein expression analysis. In addition, the blood was collected from the right ventricle using a heparin‐containing syringe with a needle at the time of death.

### Echocardiography

At the day before the sacrifice, Doppler analysis was performed to ensure that pathologic cardiac hypertrophy was induced. The internal diameter and wall thickness of left ventricle (LV) were assessed by echocardiography 19. Hearts of the killed mice were dissected and weighted to compare heart weight/bodyweight (mg/kg) ratios in mice. Mice was anaesthetized with isoflurane, echocardiography was performed by SONOS 5500 ultrasound (Philips Electronics, Amsterdam, the Netherlands) with a 15‐MHz linear array ultrasound transducer. The LV was assessed in both parasternal long‐axis and short‐axis views at a frame rate of 120 Hz. End‐systole or end‐diastole was defined as the phase in which the smallest or largest area of LV respectively. LV posterior wall (LVPW) and LV internal dimension (LVID) were measured from LV M‐model tracing with a sweep speed of 50 mm/sec. at the mid‐papillary muscle level.

### Real‐time quantitative PCR

Cells or heart tissues (20–50 mg) were homogenized in TRIZOL (Invitrogen) for extraction of RNA according to each manufacturer's protocol. Both reverse transcription and quantitative PCR were carried out using a two‐step M‐MLV Platinum SYBR Green qPCR SuperMix‐UDG kit (Invitrogen). Eppendorf Mastercycler eprealplex detection system (Eppendorf, Hamburg, Germany) was used for qPCR analysis. The primers of genes including atrial natriuretic peptide (ANP), B type natriuretic peptide (BNP), α‐MyHC, β‐MyHC, skeletal actin (SKA) and β‐actin were synthesized from Invitrogen (Shanghai, China). The primer sequences used were listed in the Table S1. The amount of each gene was determined and normalized to the amount of β‐actin.

### Western immunoblot analysis

Cells or heart tissues (30–50 mg) were lysated. Fifty micrograms of lysates were separated by 10% SDS‐PAGE and electrotransferred to a PVDF membrane. Each membrane was pre‐incubated for 1.5 hrs at room temperature in Tris‐buffered saline, pH 7.6, containing 0.05% Tween 20 and 5% non‐fat milk. Each PVDF membrane was incubated with specific antibodies. Immunoreactive bands were then detected by incubating with a secondary antibody conjugated with horseradish peroxidase and visualizing using enhanced chemiluminescence reagents (Bio‐Rad, Hercules, CA, USA). The amounts of the proteins were analysed using Image J analysis software version 1.38e and normalized to their respective control.

### Heart histopathology

Hearts were fixed in 4% paraformaldehyde and embedded in paraffin. The paraffin sections (5 μm) were stained with haematoxylin and eosin. To estimate the extent of damage, the specimen was observed under a light microscope (×200 amplification; Nikon, Tokyo, Japan).

### Immunohistochemistry

After deparaffinization and rehydration, 5 μm heart sections were treated with 3% H_2_O_2_ for 30 min. and with 1% BSA in PBS for 30 min. Slides were incubated overnight at 4°C with primary antibody (1:50) then incubated with secondary antibody (1:100; Santa Cruz Biotech) for 1 hr and DAB (A: B = 1: 20) for 5 min. at room temperature. At last the cell nuclei were stained with haematoxylin for 5 min., the sections were dehydrated and the images were viewed by a light microscope (×200 amplification; Nikon).

### Masson staining for fibrosis

Tissues were fixed in paraformaldehyde solution and embedded in paraffin. Paraffin sections (5 μm) of the heart tissues were stained with Masson to evaluate the fibrosis content. The stained sections then were viewed by light microscope (×200 amplification; Nikon).

### Statistical analysis

All data represent three independent experiments and are expressed as means ± S.E.M. All statistical analyses were performed with GraphPad Pro. Prism 5.0 (GraphPad, San Diego, CA, USA). Student's *t*‐test and two‐way anova were employed to analyse the differences between sets of data. A *P*‐value < 0.05 was considered significant.

## Results

### AG1478, 542 and 543 inhibits the activation of EGFR and its downstream signalling induced by EGF and Ang II in H9c2 cells

**Figure 2:**
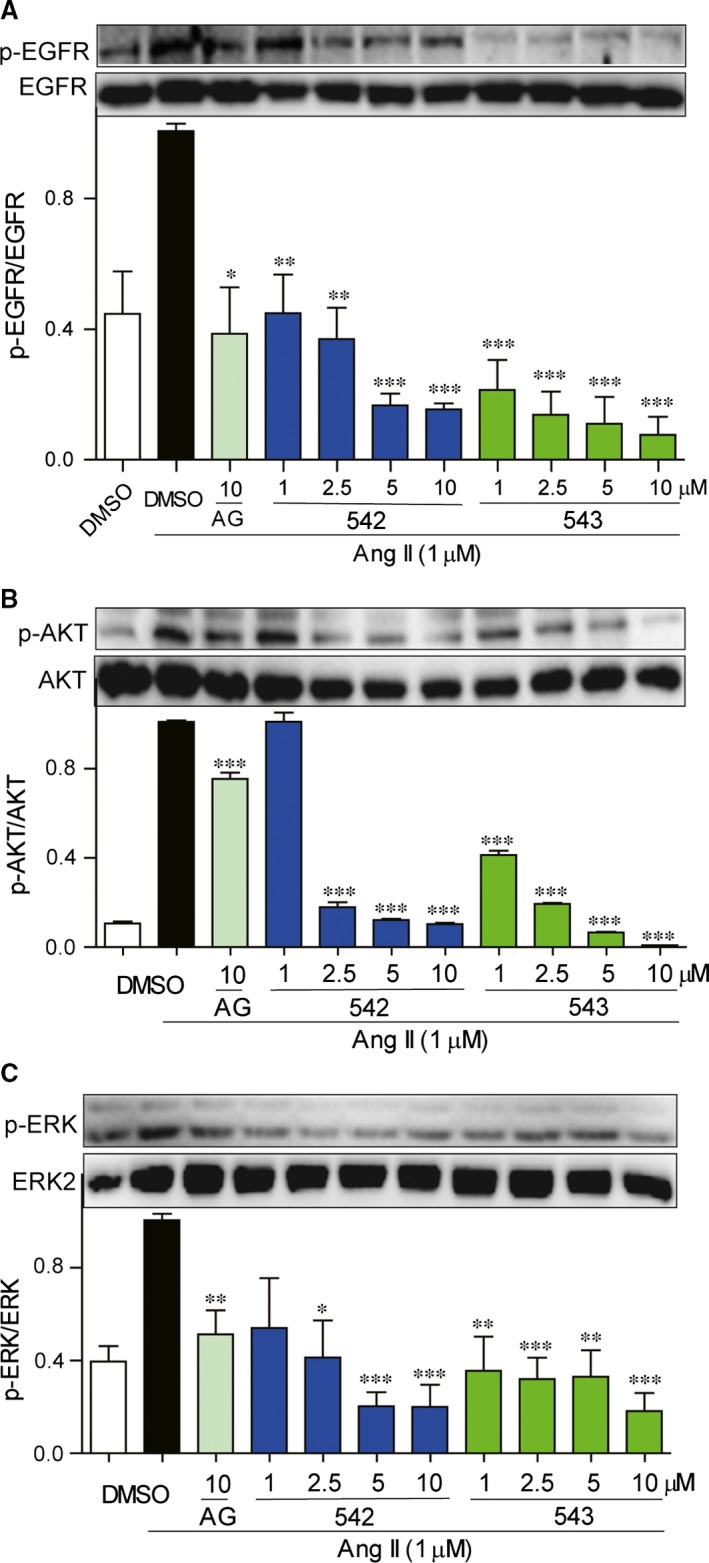
EGFR inhibitors suppressed Ang II‐induced EGFR signalling pathway activation in H9c2 cells. (**A**–**C**) H9c2 cells were pre‐treated with AG1478 (10 μΜ), 542 (1, 2.5, 5 or 10 μΜ) or 543 (1, 2.5, 5 or 10 μΜ) for 2 hrs, and followed incubation of Ang II (1 μΜ) for 15 min. The cell lysates were collected and p‐EGFR/EGFR (**A**), p‐AKT/AKT (**B**) and p‐ERK/ERK (**C**) were detected by Western blot analysis. The columns show the normalized optical density for data from three independent experiments. *P < 0.05, **P < 0.01, ***P < 0.001 *versus* Ang II group.

We detected the effects of these inhibitors on EGFR signalling induced by EGF in H9c2 cells. Western bolt analysis showed that cardiac H9c2 cells could express high‐level EGFR and ERK/AKT proteins and EGF remarkably activated EGFR pathway (Fig. 1B–D). All of three inhibitors significantly decrease EGFR phosphorylation (Fig. 1B) and downstream AKT (Fig. 1C) and ERK (Fig. 1D) phosphorylation induced by EGF. We then detected the effects of Ang II on EGFR signalling in cardiac H9c2 cells. H9c2 cells were pre‐treated with AG1478 (10 μΜ), 542 (1, 2.5, 5 or 10 μΜ) or 543 (1, 2.5, 5 or 10 μΜ) for 2 hrs, followed by the stimulation by Ang II (1 μΜ) for 15 min. As shown in Figure 2A–C, the levels of EGFR, AKT, and ERK phosphorylation were significantly increased by Ang II treatment, and our EGFR inhibitors dose‐dependently suppressed the Ang II‐induced activation of EGFR and ERK/AKT in H9c2 cells.

### EGFR inhibitors 542 and 543 attenuate AngII‐induced hypertrophy of H9c2 cells

**Figure 3:**
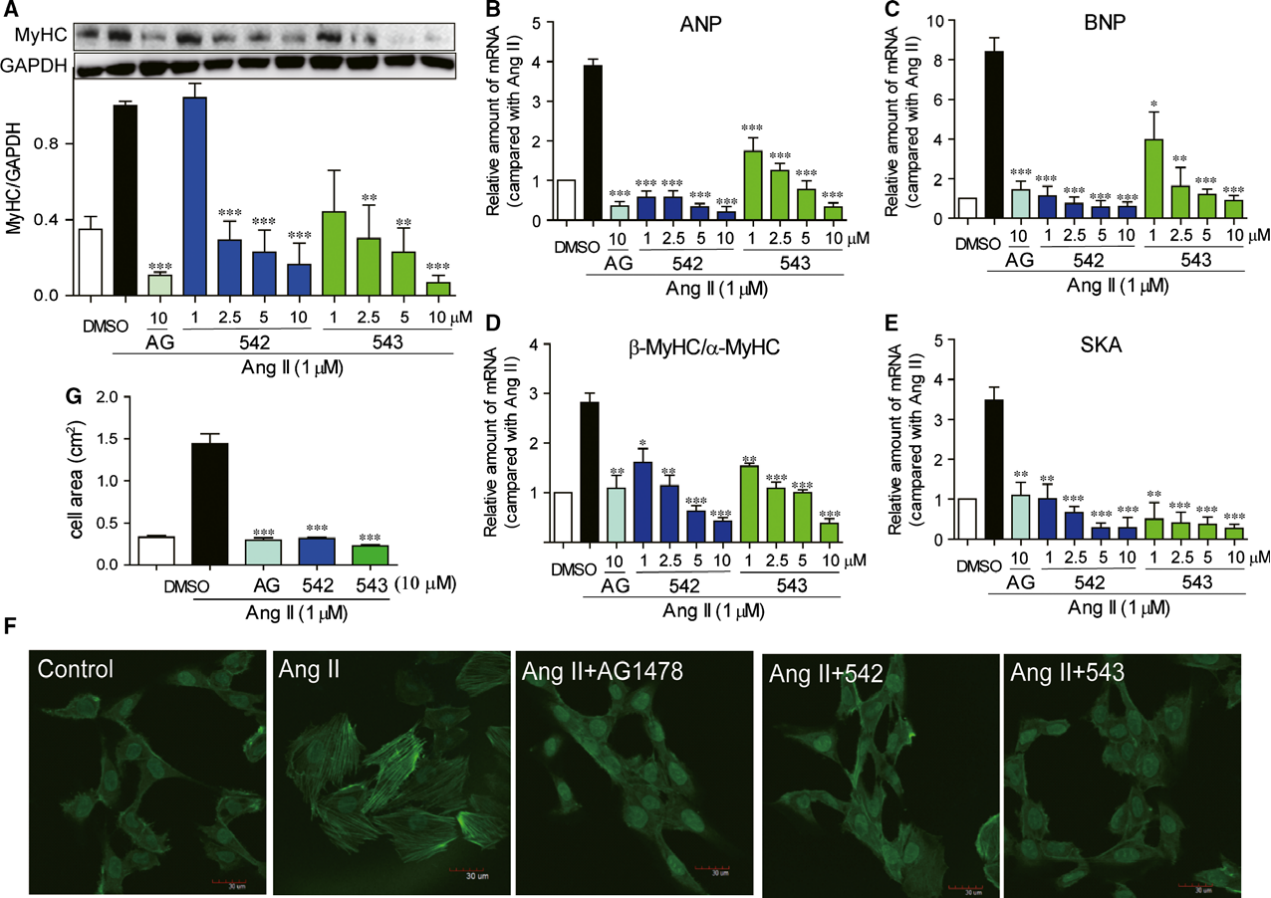
EGFR inhibitors attenuated Ang II‐induced cardiac hypertrophy in H9c2 cells. (**A**) H9c2 cells were pre‐treated with AG1478 (10 μΜ), 542 (1, 2.5, 5 or 10 μΜ) or 543 (1, 2.5, 5 or 10 μΜ) for 2 hrs, and followed incubation of Ang II (1 μΜ) for 12 hrs. The cell lysates were collected and MyHC was detected by Western blot analysis. (**B**–**E**) H9c2 cells were pre‐treated with AG1478 (10 μΜ), 542 (1, 2.5, 5 or 10 μΜ) or 543 (1, 2.5, 5 or 10 μΜ) for 2 hrs, and followed incubation of Ang II (1 μΜ) for 6 hrs. The mRNA levels of ANP (**B**), BNP (**C**), β/α‐MyHC (**D**) and SKA (**E**) were detected by RT‐qPCR assay. (**F** and **G**) H9c2 cells were pre‐treated with AG1478 (10 μΜ), 542 (10 μΜ) or 543 (10 μΜ) for 2 hrs, and followed incubation of Ang II (1 μΜ) for 24 hrs. The cell areas were detected by immunofluorescence assay. The columns show the normalized optical density for data from three independent experiments. *P < 0.05, **P < 0.01, ***P < 0.001 *versus* Ang II group.

We examined whether EGFR inhibitors AG1478, 542 and 543 could reduce AngII‐induced expression of cardiac hypertrophic markers ANP, BNP, β/α‐MyHC and SKA and morphological expand in H9c2. H9c2 cells were pre‐treated with compounds for 2 hrs and then treated with Ang II (1 μΜ) for 6 hrs. Western blot, RT‐qPCR and immunofluorescence staining assays were used to detect the hypertrophic markers. As shown in Figure 3, the protein expression of MyHC (Fig. 3A), and the mRNA level of ANP (Fig. 3B), BNP (Fig. 3C), β/α‐MyHC (Fig. 3D) and SKA (Fig. 3E) were significantly increased in H9c2 cells exposed to Ang II, while pre‐treatment with either 542 or 543 dose‐dependently inhibited the overexpression of these hypertrophic genes. Subsequently, fluorescence microscopic study showed that all of three inhibitors markedly reduced the Ang II‐induced hypertrophy in cardiac H9c2 cells (Fig. 3F and G). These data suggested that EGFR inhibitors significantly attenuated Ang II‐induced cardiac hypertrophy *in vitro*.

### EGFR silencing prevents AngII‐induced hypertrophy in H9c2 cells

**Figure 4:**
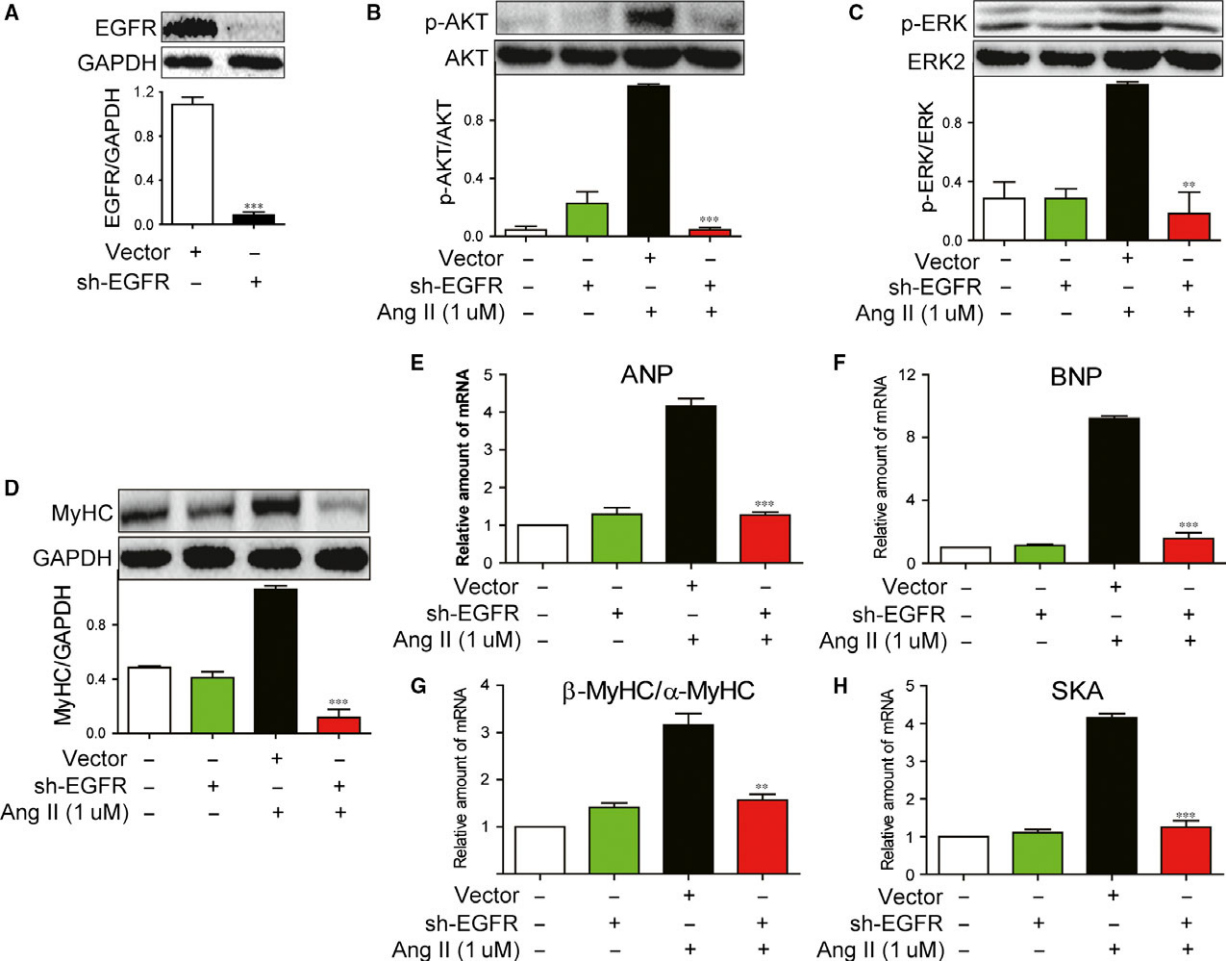
EGFR shRNA inhibited Ang II‐induced EGFR signalling pathway activation and cardiac hypertrophy in H9c2 cells. (**A**) H9c2 cells were transfected with EGFR or vehicle shRNA for 48 hrs, the expression of EGFR was detected by Western blot analysis. (**B** and **C**) H9c2 cells were transfected with EGFR or vehicle shRNA for 48 hrs, and followed incubation of Ang II (1 μΜ) for 15 min. The cell lysates were collected and p‐AKT/AKT (**B**) and p‐ERK/ERK (**C**) were detected by Western Blot analysis. (**D**) H9c2 cells were transfected with EGFR or vehicle shRNA for 48 hrs, and followed incubation of Ang II (1 μΜ) for 12 hrs. The cell lysates were collected and MyHC was detected by Western Blot analysis. (**E**–**H**) H9c2 cells were transfected with EGFR or vehicle shRNA for 48 hrs, and followed incubation of Ang II (1 μΜ) for 6 hrs. The mRNA levels of ANP (**E**), BNP (**F**), β/α‐MyHC (**G**) and SKA (**H**) were detected by RT‐qPCR assay. The columns show the normalized optical density for data from three independent experiments. **P < 0.01, ***P < 0.001 *versus* Ang II group.

To exclude possible non‐specific inhibition by the pharmacological inhibitors, H9c2 cells also were transfected with an EGFR shRNA‐containing plasmid and then exposed to Ang II for different times. Figure 4A showed that EGFR shRNA transfection could significantly silence the EGFR expression in H9c2 cells. The silencing efficiency was further confirmed by the fact that EGFR shRNA significantly prevented the Ang II‐induced phosphorylation of downstream AKT (Fig. 4B) and ERK1/2(Fig. 4C). Importantly, Figure 4D showed that EGFR silencing significantly prevented the Ang II‐induced expression of MyHC protein, suggesting the function of EGFR in regulating the MyHC expression. The mediating role of EGFR was further confirmed by real‐time qPCR analysis, which showed that the Ang II‐increased mRNA levels of four hypertrophic genes including ANP, BNP, β/α‐MyHC and SKA were also remarkably prevented by EGFR knockdown in EGFR shRNA‐transfected H9c2 cells (Fig. 4E–H). These data further validate that EGFR plays a critical role in mediating cardiac hypertrophy induced by Ang II.

### EGFR inhibitors attenuate EGF‐induced hypertrophy in H9c2 cells

**Figure 5:**
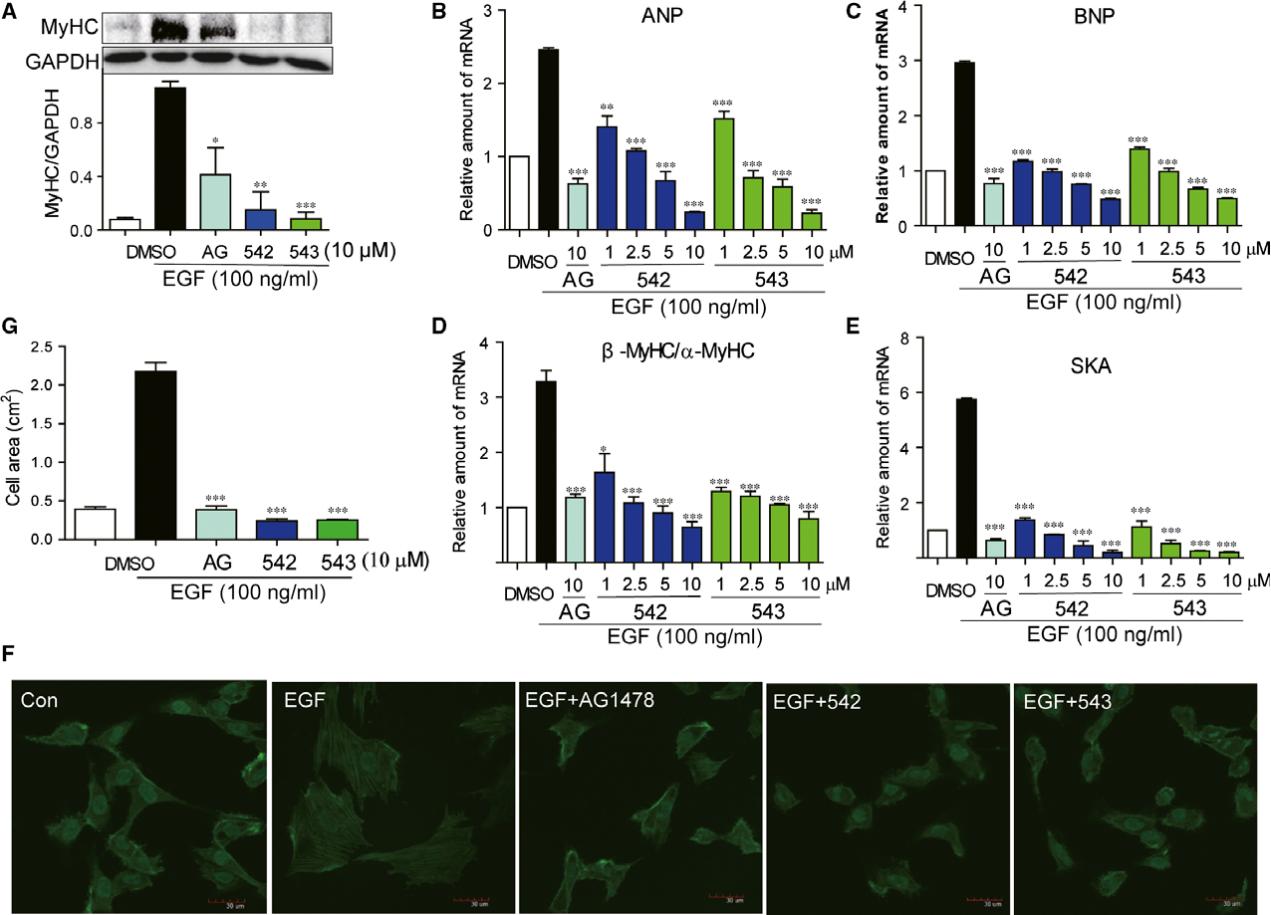
EGFR inhibitors suppressed EGF‐induced cardiac hypertrophy in H9c2 cells. (**A**) H9c2 cells were pre‐treated with AG1478 (10 μΜ), 542 (10 μΜ) or 543 (10 μΜ) for 2 hrs, and followed incubation of EGF (100 ng/ml) for 12 hrs. The cell lysates were collected and MyHC was detected by Western blot analysis. (**B**–**E**) H9c2 cells were pre‐treated with AG1478 (10 μΜ), 542 (1, 2.5, 5 or 10 μΜ) or 543 (1, 2.5, 5 or 10 μΜ) for 2 hrs, and followed incubation of EGF (100 ng/ml) for 6 hrs. The mRNA levels of ANP (**B**), BNP (**C**), β/α‐MyHC (**D**) and SKA (**E**) were detected by RT‐qPCR assay. (**F** and **G**) H9c2 cells were pre‐treated with AG1478 (10 μΜ), 542 (10 μΜ) or 543 (10 μΜ) for 2 hrs, and followed incubation of EGF (100 ng/ml) for 24 hrs. The cells areas were detected by immunofluorescence assay. The columns show the normalized optical density for data from three independent experiments. *P < 0.05, **P < 0.01, ***P < 0.001 *versus* Ang II group.

Although EGFR activation mediates Ang II‐induced hypertrophy, it is still unknown if EGFR activation could directly induce hypertrophy in H9c2 cells. Thus, we detected the pro‐hypertrophic effects of EGF, a direct ligand of EGFR. Figure 5A showed that incubation with EGF (100 ng/ml) for 6 hrs significantly increased MyHC protein expression in H9c2 cells. Similar results were observed in the mRNA expression of four hypertrophic markers ANP, BNP, β/α‐MyHC and SKA (Fig. 5B–E). These overexpressions of pro‐hypertrophic genes induced by EGF were significantly inhibited by AG, 542 and 543 in dose‐dependent manner. Fluorescence microscopic analysis also revealed that EGF treatment significantly increased the morphological expansion of H9c2 cells and all of three inhibitors reversed the EGF‐induced pathological hypertrophy (Fig. 5F and G). The data indicate that EGFR activation may be a direct and independent inducer for cardiac hypertrophy.

### EGFR inhibitor 542 attenuates cardiac hypertrophy *in vivo*

**Figure 6:**
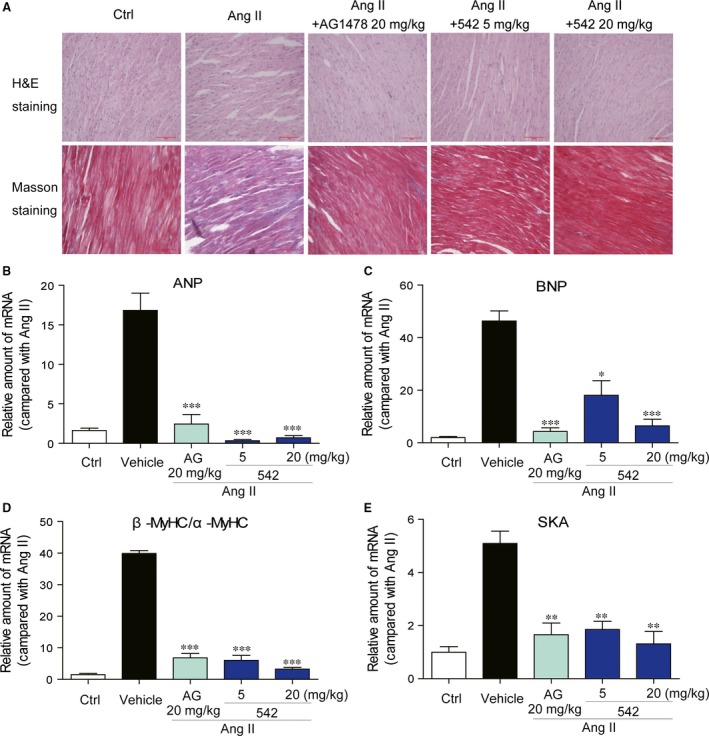
Administration of 542 attenuated Ang II‐induced cardiac hypertrophy *in vivo*. Male C57BL/6 mice were given a subcutaneous injection of AngII (0.5 mg/kg/day) for 2 weeks and, simultaneously, were treated with EGFR inhibitors by daily gavage. After the finial administration, blood samples and heart tissues were collected for further experiments. (**A**) A representative haematoxylin and eosin and Masson staining for heart tissue from six mice studied in each group is shown. (**B**–**E**) Heart tissues from mice were individually processed for RNA extraction and RT‐qPCR. The mRNA levels of ANP (**B**), BNP (**C**), β/α‐MyHC (**D**) and SKA (**E**) were determined (*n* = 7 per group, *P < 0.05, **P < 0.01, ***P < 0.001 *versus* Ang II group.).

Generally, compound 542 showed slightly stronger EGFR inhibition and anti‐hypertrophy activity than 543. Thus, compound 542 as well as AG1478 was chosen for the *in vivo* study to confirm the protective effect of EGFR inhibition on Ang II‐induced cardiac hypertrophy. C57BL/6 mice are given a subcutaneous injection of Ang II for 2 weeks, and simultaneously, are treated with EGFR inhibitors by daily gavage. Firstly, we detected the cardiac function of the mice in each group by transthoracic echocardiograph. As shown in Table 1, Ang II injection significantly induced a cardiac hypertrophy and impaired the functioning of the diastolic and systolic LV, which were largely attenuated by the treatment with 542 at 20 mg/kg for 2 weeks. After mice killed, we examined the fibrosis and histopathology in mouse hearts. Haematoxylin and eosin staining showed that Ang II‐stimulated hearts displayed structural abnormalities, including broken fibres, deranged cellular structures. These abnormalities were significantly attenuated by administration with AG1478 or 542 (Fig. 6A). Masson staining revealed a marked collagen accumulation and fibrosis in the heart of Ang II‐treated mice, while administration of 542 dose‐dependently reduced the degree of cardiac fibrosis (Fig. 6A). We further determined whether EGFR inhibitors affected the expression of cardiac hypertrophic markers including ANP, BNP, β/α‐MyHC and SKA.

**Table 1:** Echocardiographic parameters of the experimental mice

	Control	Vehicle	AG1478	542	
				5 mg/kg	20 mg/kg
		AngII			
HW (mg)/BW (g)	4.91 ± 0.42*	6.19 ± 0.39	5.68 ± 0.62	5.09 ± 0.38	5.01 ± 0.26*
VS, mm	0.85 ± 0.07***	1.09 ± 0.04	0.95 ± 0.02***	0.88 ± 0.05***	0.80 ± 0.08***
LVPW, mm	0.89 ± 0.04***	1.02 ± 0.04	0.88 ± 0.03***	0.83 ± 0.07***	0.81 ± 0.06***
LVIDd, mm	3.43 ± 0.31***	2.59 ± 0.19	2.90 ± 0.26	2.78 ± 0.07	2.82 ± 0.09*
LVIDs, mm	1.78 ± 0.29*	1.37 ± 0.16	1.57 ± 0.21	1.42 ± 0.07	1.45 ± 0.14
EF%	48.63 ± 3.98	46.48 ± 3.37	46.18 ± 4.36	49.50 ± 2.10	49.80 ± 4.04
FS%	85.40 ± 3.58	84.12 ± 2.59	83.32 ± 4.01	86.38 ± 1.64	86.40 ± 3.46
HR	372.00 ± 29.83	369.33 ± 28.45	345.83 ± 70.87	356.67 ± 40.61	403.17 ± 33.50

*P < 0.05, ***P < 0.001 *versus* Ang II‐treated group.

*n* = 7 per group.

LVPW: left ventricle posterior wall; LVIDs: systolic left ventricle internal dimension; LVIDd: diastole left ventricle internal dimension; HW: heart weight; BW: bodyweight.

**Figure 7:**
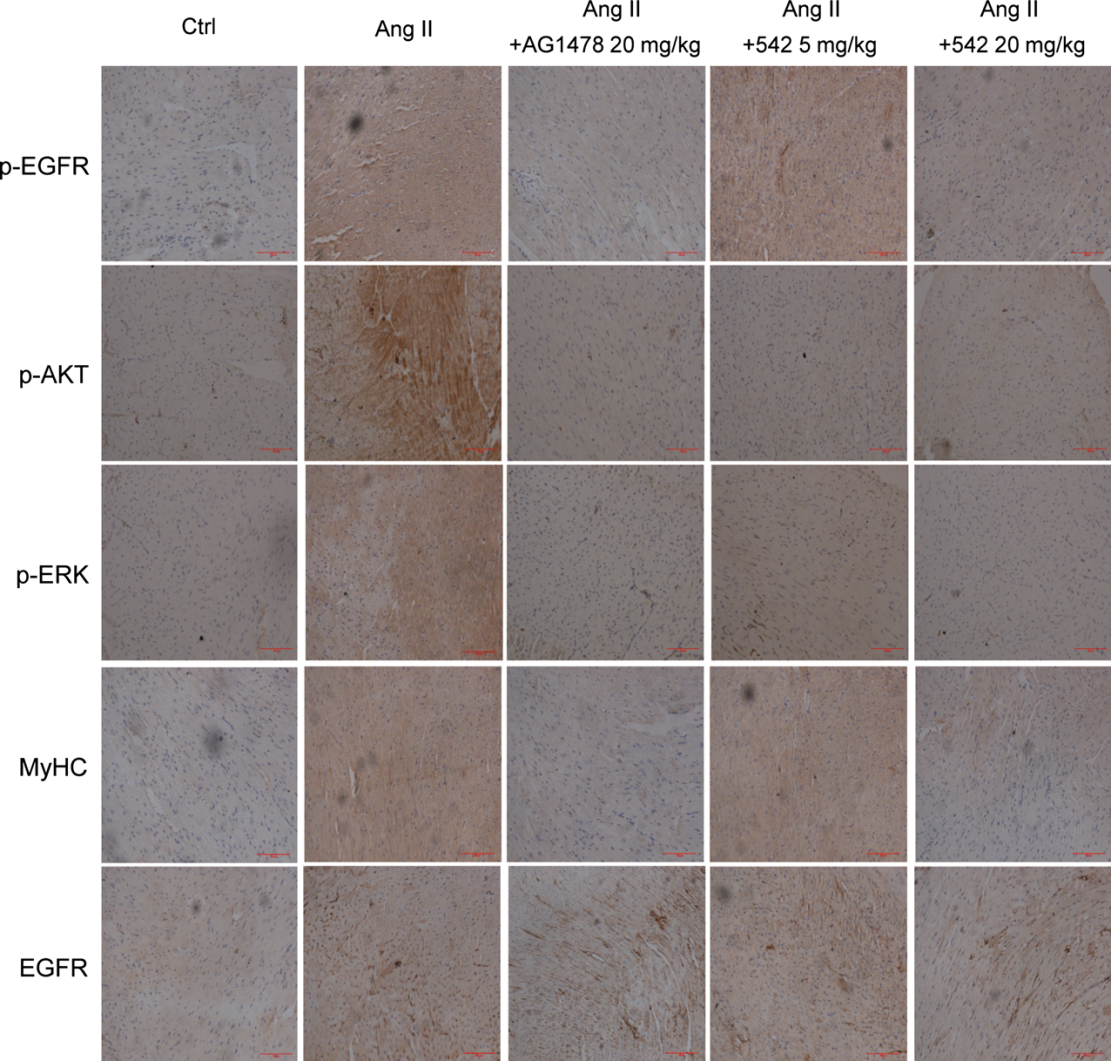
Administration of 542 inhibited Ang II‐induced EGFR signalling pathway activation and MyHC expression *in vivo*. Heart tissues were sectioned at 5 μm, and the slides were processed for immunohistochemistry assay to detect the expression of p‐EGFR, p‐AKT, p‐ERK, MyHC and EGFR (*n* = 7 in each group).

Real‐time qPCR analysis revealed significant increases in the expression of these four hypertrophic genes (Fig. 6B–E) in the Ang II‐treated hearts, but these changes were significantly blocked by the administration of either AG1478 or 542 at both dosages. Finally, we detected the activation of EGFR signalling pathway in mouse hearts. The immunohistochemical staining showed that Ang II injection markedly increased the levels of p‐EGFR, p‐ERK and p‐AKT in mouse hearts, while AG1478 and 542 at 20 mg/kg significantly inhibited the cardiac EGFR signalling pathway activation (Fig. 7). In addition, the immunohistochemical staining for cardiac MyHC confirmed the anti‐hypertrophic effect of EGFR inhibitors in Ang II‐treated mice (Fig. 7).

### Effect of PP2 on AngII‐induced EGFR phosphorylation

**Figure 8:**
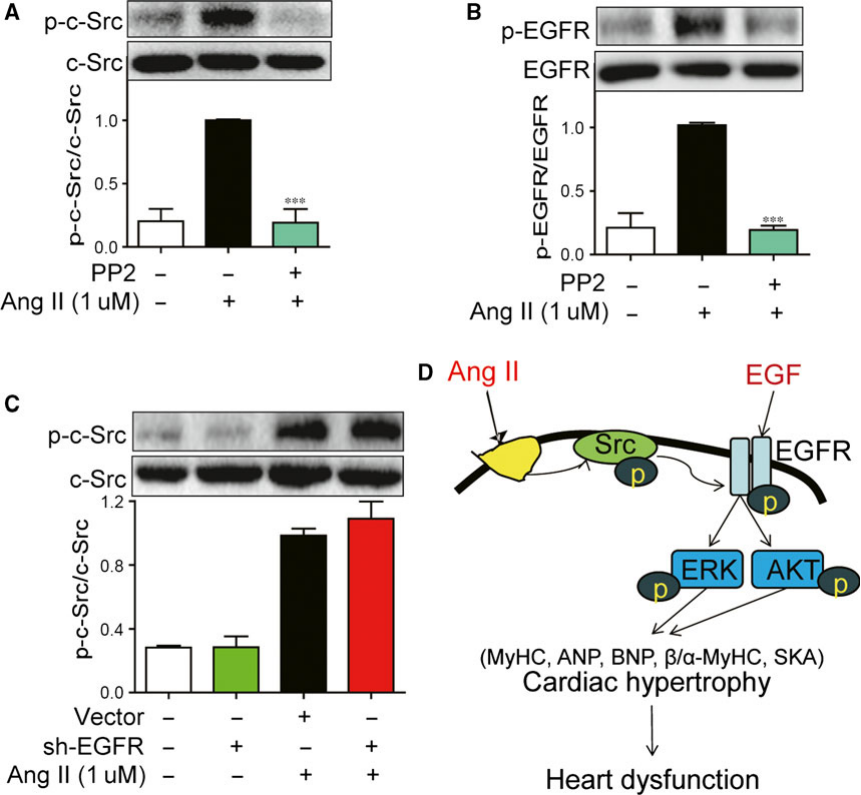
PP2 inhibited Ang II‐induced EGFR transactivation in H9c2 cells. (**A** and **B**) H9c2 cells were pre‐treated with PP2 (10 μM) for 2 hrs and followed incubation of Ang II (1 μΜ) for 15 min. The cell lysates were collected and p‐c‐Src/Src (**A**) and p‐EGFR/EGFR (**B**) were detected by Western blot analysis. (**C**) H9c2 cells were transfected with EGFR or vehicle shRNA for 48 hrs and followed incubation of Ang II (1 μΜ) for 15 min. The cell lysates were collected and p‐c‐Src/c‐Src was detected by Western blot analysis. ***P < 0.001 *versus* Ang II group. (**D**) A schematic diagram depicting the underlying mechanism of Ang II‐induced cardiac hypertrophy.

It is still unknown how Ang II activates EGFR. A growing body of evidence has suggested that c‐Src plays an important role in regulating EGFR transactivation 20. A specific small‐molecule c‐Src inhibitor (PP2) was used for this study. The results in Figure 8A and B showed that PP2 almost totally inhibited the phosphorylation of both p‐c‐Src and EGFR induced by Ang II, indicating that c‐Src may mediate AngII‐induced EGFR phosphorylation. We also used EGFR shRNA transfection to test the possible effect of EGFR on Ang II‐induced c‐Src phosphorylation. Figure 8C revealed that EGFR silencing was not able to affect Ang II‐induced c‐Src activation, validating that c‐Src should be the upstream regulator of EGFR in Ang II cascade.

## Discussion

In the current study, we demonstrate that EGFR inhibitors have protective effects against Ang II‐induced cardiac hypertrophy, validating a critical role of EGFR in mediating cardiac hypertrophy. Epidermal growth factor receptor, a receptor tyrosine kinase, is endogenously expressed in numerous cell types and is an important factor in the control of many fundamental cellular processes, including the cell migration, cell cycle, cell metabolism and survival, in addition to cell proliferation and differentiation 21. Epidermal growth factor receptor and its ligands are frequently up‐regulated in human cancers 21. Consequently, blocking the proliferative effects of EGF receptor activation has potential therapeutic applications in cancer and aberrant tissue growth. A number of small‐molecule specific inhibitors of EGFR have been developed for cancer treatment and more than six inhibitors have been used in clinical.

It has been observed that a modest number of patients, suffering from both malignancies and type 2 diabetes, were successfully treated not only for their malignancies but also for diabetes when given some tyrosine kinase inhibitors, indicating that tyrosine kinases including EGFR also mediated the metabolic diseases 22, 23, 24, 25. Recently, the role of EGFR in maintaining organ and cellular homoeostasis is becoming more and more evident especially in the metabolic and cardiovascular systems 26. The EGFR could be expressed in vascular smooth muscle cells, cardiomyocytes and endothelial cells, and all of these cells also secrete EGFR ligands 26. Our data also showed that both H9c2 cells and heart tissue have high EGFR expression level (Figs 1, 2 and 7). Inhibition of EGFR has been reported to be beneficial in improving insulin resistance and type 2 diabetes, attenuating diabetic nephropathy and kidney fibrosis, inhibiting cardiac hypertrophy and heart failure, and reducing atherosclerosis and elevated arterial blood pressure in experimental animal models 9, 13, 26, 27. However, the precise molecular mechanisms that account for the effect of EGFR inhibition on CVDs are still unclear.

Pathological cardiac hypertrophy is a consequence of the maladaptive alterations and an important pathological process in diverse cardiac diseases. The EGFR pathway activation has been shown to contribute to the development of cardiac hypertrophy. Abrogation of EGFR kinase activity by selective pharmacological inhibitors or antisense oligonucleotides significantly protects against cardiac hypertrophy 19, 28. Renin–angiotensin system activation mediates and contributes a lot to the pathogenesis of a variety of CVDs. Ang II has an important role in cardiovascular and renal pathophysiology 3. It has been shown that sustained Ang II infusion induced vascular injury and cardiac hypertrophy 3. Several signalling molecules have been implicated in the Ang II‐induced hypertrophic response, including MAPKs, tyrosine kinases such as, and PI3K/AKT, and some receptor tyrosine kinases 29, 30. It has been demonstrated that transactivation of EGFR by Ang II led to vascular resistance and high blood pressure, and EGFR inhibition by AG1478 has been shown to prevent Ang II‐induced hypertension 20. Our results suggested that the EGFR inhibition by either three small‐molecule inhibitors or shRNA silencing could attenuate cardiac hypertrophy in Ang II‐treated H9c2 cells and primary cardiomyocytes. Administration with AG and 542 also significantly reversed Ang II‐induced cardiac hypertrophy and heart dysfunctions in mice (Fig. 6). These results indicated that EGFR play a critical role in mediating the induction of cardiac hypertrophy by Ang II, which may be one of mechanisms by which EGFR inhibition attenuates a variety of CVDs.

It is well known that EGFR transmits signals to prominent downstream pathways, such as MAPKs and PI3K/Akt pathways. PI3K/AKT signalling pathway has been implicated in the Ang II‐induced hypertrophic response. In the heart, short‐term AKT activation promotes physiological hypertrophy, whereas long‐term AKT activation induced pathological hypertrophy 31. Among the MAPKs, ERK1/2 has been considered as the essential regulator of a hypertrophic response, although JNK and p38 were also recently examined in regulating cardiac hypertrophy 32, 33. Downstream targets of ERK and PI3K/Akt include transcription factors such as NF‐kB, AP‐1 and Smad, which have been shown to regulate inflammation and fibrosis respectively 19, 34, 35. And, mounting evidence has strongly suggested that inflammation and fibrosis play a key role in the development of cardiac hypertrophy and heart failure 36, 37. Our data clearly demonstrated that ERK1/2 and AKT were all significantly phosphorylated both in Ang II‐treated cardiomyocytes and mice, accompanied by hypertrophy and fibrosis. Three EGFR inhibitors markedly inhibited both ERK1/2 and AKT activation *in vivo* and *in vitro* in response to hypertrophic stimuli of Ang II. These findings confirmed AKT and ERK as pivotal regulators of cardiac hypertrophy and suggested that the pro‐hypertrophic effect of EGFR in heart may be mediated by its downstream AKT and ERK signalling pathways.

The mechanism by which Ang II activates EGFR signalling is still unclear. Activation of EGFR occurs either by binding with ligands, such as EGF and heparin bound‐EGF, or by transactivation. The c‐Src has been shown to be activated by Ang II for downstream signalling transduction 27. In addition, it has been demonstrated that c‐Src‐dependent transactivation of EGFR and Akt/MAPKs is essential for COX‐2 up‐regulation in endothelial cells 38. In the present study, we showed that the c‐Src inhibitor PP2 significantly blocked Ang II‐induced EGFR phosphorylation, while EGFR shRNA had no effect on Ang II‐induced c‐Src phosphorylation. These data indicate that c‐Src activation may mediate Ang II‐induced EGFR transactivation (Fig. 8D). Another important finding is that cardiac hypertrophy mediated by EGFR activation is Ang II‐independent, as demonstrated by the fact that EGF stimulation alone induced cardiac hypertrophy in H9c2 cells (Fig. 5). This suggests that EGFR auto‐activation or EGFR overexpression resulted from genetic mutation that occurs frequently in cancer genesis and development may be also a risk for cardiac diseases, despite that no tumourigenesis was observed in heart. Further study should consider and confirm EGFR as a marker and a therapeutic target for cardiac diseases, and even as a cross‐linker for cardiac diseases and cancer, especially EGFR‐positive cancer.

In addition, the data obtained from H9c2 cell line was confirmed by primary cardiomyocyte. Isolation of primary neonatal rat cardiomyocytes was performed as described in Supplementary File. As shown in the supplementary Figure S1, primary cardiomyocytes express a relative high amount of EGFR and EGFR inhibitors significantly blocked Ang II‐induced EGFR/AKT/ERK phosphorylation and overexpression of pro‐hypertrophic genes including MyHC, ANP and SKA, indicating that EGFR plays similar role in primary rat cardiomyocytes.

In conclusion, we demonstrate that EGFR play a key role in cardiac hypertrophy and EGFR inhibition by either small‐molecule inhibitors or genetic silencing can protect against Ang II‐induced cardiac hypertrophy. Although the role of EGFR in Ang II‐induced cardiac hypertrophy has been studied in the previous paper, in which authors silenced EGFR expression by injection of EGFR antisense oligodeoxynucleotide in Ang II‐infused rats 28, our work tested the effects of three small‐molecule EGFR inhibitors on Ang II‐induced cardiac hypertrophy in mice, which may be close to a translational study. Using both small‐molecule EGFR inhibitors and EGFR shRNA, we investigated the mechanism of EGFR activation in cardiomyocytes and concluded that c‐Src mediated the EGFR activation induced by Ang II. Based on the observations from the literature and our findings, Figure 8D depicts a model for the signalling mechanisms implicated in Ang II‐induced cardiac hypertrophy, mediated by c‐Src‐dependent transactivation of EGFR linking to ERK/AKT pathways in H9c2 cells. This study suggests that targeting EGFR may provide useful therapeutic strategies for cardiac diseases and these two novel EGFR inhibitors 542 and 543 may be promising for the further drug development. Although several EGFR‐specific inhibitors and antibody have been used in clinical cancer therapy, the extent to which inhibition of these molecules is clinically applicable to the prevention and treatment of cardiac diseases in human patient remains to be determined.

## Conflicts of interest

All the authors declare no competing financial interest.

## Author contribution

K.P., X.T., Y.Q., Z.X., M.S and C.Z. researched data; G.L., K.P. and J.W. contributed initial discussion of the project; X.L. and Z.L. reviewed the article; G.L. and K.P. wrote the article.

## Supporting information


**Figure S1** Effects of EGFR inhibitors in Ang II‐stimulated primary neonatal rat cardiomyocytes.
**Table S1** Primer sequences for real‐time quantitative PCR.
