# Supplementary material for: Novel EGFR inhibitors attenuate cardiac hypertrophy induced by angiotensin II
Source: J Cell Mol Med. 2016 Jan 14;20(3):482–94. doi: 10.1111/jcmm.12763 (PMC4759478; doi:10.1111/jcmm.12763)
Supplement: Supplementary file 1 — Figure S1 Effects of EGFR inhibitors in Ang II‐stimulated primary neonatal rat cardiomyocytes. Table S1 Primer sequences for real‐time quantitative PCR. [file JCMM-20-482-s001.doc]

**Supplementary file**

**Novel EGFR inhibitors attenuate cardiac hypertrophy induced by angiotensin II**

Kesong Peng^a,*^, Xinqiao Tian^b,*^, Yuanyuan Qian^a^, Melissa Skibba^a^, Chunpeng Zou^b^, Zhiguo Liu^a^, Jingying Wang^a^, Zheng Xu^a^, Xiaokun Li^a,#^, Guang Liang^a,#^

^a^ Chemical Biology Research Center, School of Pharmaceutical Science, Wenzhou Medical University, Wenzhou, Zhejiang, China

^b^ Department of Ultrasonography, the 2^th^ Affiliated Hospital of Wenzhou Medical University, Wenzhou, Zhejiang, China

**AG1478, 542, and 543 inhibits the EGFR/AKT/ERK activation and pro-hypertrophic gene expression in primary neonatal rat cardiomyocytes**

**Isolation and culture of primary cardiomyocytes**

Primary cultures of neonatal rat cardiomyocytes were performed using a method published in our previous publication (Diabetes. 2014;63:3497-511.). Cells were incubated with a DMEM medium that contained 5.5 mmol/L of D-glucose.

**Results**

All data obtained using primary cardiomyocytes are shown in Figure S1. As indicated in Figure S1A, primary cardiomyocytes express a relative high amount of EGFR and Ang II treatment significantly increased the phosphorylation of EGFR in primary cardiomyocytes, while these alterations were markedly reversed by the pre-treatment with AG1478 (10μM), 542 (10μM), or 543 (10μM). Furthermore, we determined the effects of EGFR inhibitors on Ang II-induced AKT/ERK phosphorylation and pro-hypertrophic gene expression in primary cardiomyocytes. As shown in Figure S1B-F, EGFR inhibitors significantly inhibited Ang II-induced AKT/ERK phosphorylation and expressions of MyHC, ANP, and SKA. Overall, these data indicates that EGFR plays similar role in primary rat cardiomyocytes.

**Figure S1.** Effects of EGFR inhibitos in Ang II-stimulated primary neonatal rat cardiomyocytes. (A-B) H9c2 cells were pretreated with AG1478 (10 μΜ), 542 (10 μΜ), or 543 (10 μΜ) for 2h, and followed incubation of Ang II (1 μΜ) for 15min. The cell lysates were collected and p-EGFR/EGFR (A) and p-AKT/p-ERK/GAPDH (B) were detected by Western Blot analysis. (C) H9c2 cells were pretreated with AG1478 (10 μΜ), 542 (10 μΜ), or 543 (10 μΜ) for 2h, and followed incubation of Ang II (1 μΜ) for 12h. The cell lysates were collected and MyHC/GAPDH was detected by Western Blot analysis. (D-F) H9c2 cells were pretreated with AG1478 (10 μΜ), 542 (10 μΜ) or 543 (10 μΜ) for 2h, and followed incubation of Ang II (1 μΜ) for 6 h. The mRNA levels of ANP (D), β/α-MyHC (E), and SKA (F) were detected by RT-qPCR assay. The columns show data from three independent experiments. *P <0.05, **P <0.01 vs Ang II group.

**Table S1.** Gene primer sequences for real-time quantitative PCR analysis.

| **Table S1. Primer sequences for real-time quantitative PCR.** | | | |
| --- | --- | --- | --- |
| Source | Gene | Sequence 5′–3′ (forward) | Sequence 5′–3′ (reverse) |
| Rat | ANP | GAGGAGAAGATGCCGGTAG | CAGAGAGGGAGCTAAGTG |
|  | BNP | TTCCGGATCCAGGAGAGACTT | CCTAAAACAACCTCAGCCCGT |
|  | α-MyHC | CGAGTCCCAGGTCAACAAG | AGGCTCTTTCTGCTGGACA |
|  | β-MyHC | GAGGAGAGGGCGGACATT | ACTCTTCATTCAGGCCCTTG |
|  | SKA | AGAGCACGGCATTATCAC | TCATCTTCTCACGGTTGG |
|  | β-actin | AAGTCCCTCACCCTCCCAAAAG | AAGCAATGCTGTCACCTTCCC |
| Mouse | ANP | AACCTGCTAGACCACCTGGA | TGCTTTTCAAGAGGGCAGAT |
|  | BNP | GTCAGTCGTTTGGGCTGTAAC | AGACCCAGGCAGAGTCAGAA |
|  | α-MyHC | GCCAAGACTGTCCGGAATGA | TGGAAGATCACCCGGGACTT |
|  | β-MyHC | CAAAGGCAAGGCAAAGAAAG | TCACCCCTGGAGACTTTGTC |
|  | SKA | CTGGATTCTGGCGATGGTGTA | CGGACAATTTCACGTTCAGCA |
|  | β-actin | CCGTGAAAAGATGACCCAGA | TACGACCAGAGGCATACAG |
